# Cellular Signaling of Amino Acid Metabolism in Prostate Cancer

**DOI:** 10.3390/ijms26020776

**Published:** 2025-01-17

**Authors:** Ping Yao, Shiqi Cao, Ziang Zhu, Yunru Wen, Yawen Guo, Wenken Liang, Jianling Xie

**Affiliations:** 1 School of Biology and Biological Engineering, South China University of Technology, University Town, Guangzhou 510006, China; 2 Flinders Health and Medical Research Institute, Flinders University, Bedford Park, SA 5042, Australia

**Keywords:** prostate cancer, amino acid, metabolic reprogramming, mTOR, GCN2

## Abstract

Prostate cancer is one of the most common malignancies affecting men worldwide and a leading cause of cancer-related mortality, necessitating a deeper understanding of its underlying biochemical pathways. Similar to other cancer types, prostate cancer is also characterised by aberrantly activated metabolic pathways that support tumour development, such as amino acid metabolism, which is involved in modulating key physiological and pathological cellular processes during the progression of this disease. The metabolism of several amino acids, such as glutamine and methionine, crucial for tumorigenesis, is dysregulated and commonly discussed in prostate cancer. And the roles of some less studied amino acids, such as histidine and glycine, have also been covered in prostate cancer studies. Aberrant regulation of two major signalling pathways, mechanistic target of rapamycin (mTOR) and general amino acid control non-depressible 2 (GCN2), is a key driver of reshaping the amino acid metabolism landscape in prostate cancer. By summarising our current understanding of how amino acid metabolism is modulated in prostate cancer, here, we provide further insights into certain potential therapeutic targets for managing prostate cancer through metabolic interventions.

## 1. Introduction to Prostate Cancer (PCa)

PCa ranks among the top five cancers in terms of global new cancer cases, and it is the second most prevalent malignant tumour and the leading cause of cancer-related mortality in males [1]. The incidence of this cancer markedly rises with age [2], and the prognosis varies depending on the timeliness of diagnosis and intervention. For patients with distant metastatic PCa, the 5-year survival rate is merely 30% [3]. Given that the growth and proliferation of prostate epithelial cells are dependent on androgens [4], androgen deprivation therapy (ADT) becomes the front line of therapeutic interventions, which targets either the gonadotropin-releasing hormone or the androgen receptor (AR). While ADT can initially benefit the health condition of PCa patients, unfortunately, most of them will unavoidably progress to refractory castration-resistant prostate cancer (CRPC), which is fatal [5]. Therefore, there is still an urgent need for a comprehensive understanding of the key factors that contribute to disease progression for the next generation of targeted strategies.

Substantial advancements have been made in recent decades in understanding the molecular mechanisms that drive PCa development, especially PCa-specific metabolic alterations. PCa cells adapt to rapid growth and survival by altering the homeostasis of key metabolic processes, with glucose metabolism, lipid metabolism and amino acid metabolism (AAM) being the most frequently remodelled core pathways. In this review, we aim to summarise our current understanding of how AAM is dysregulated in PCa in comparison to physiological conditions, especially focusing on key altered genes, amino acid residues (glutamine, tryptophan, leucine, arginine, methionine, histidine, serine and glycine) and amino acid signalling pathways (mTOR and GCN2).

## 2. Amino Acid Metabolism in PCa

Amino acids play roles extending beyond mere support for rapid protein synthesis and cellular proliferation in cancer cells; they also serve as a vital resource for tumour invasion, metastasis and immune evasion. It is well established that cancer cells are heavily addicted to several key amino acids for tumorigenesis, such as glutamine, methionine and arginine [6], and a similar dependency has been observed in PCa [7,8,9,10,11]. Metabolomic analyses of PCa patient serum and tissue samples have also revealed alterations in the levels of other amino acids. For example, a declining trend for histidine in the serum of PCa patients was observed when benign hyperplasia progressed from to primary and metastatic cancer and CRPC [12]. In the following sections, we will provide an overview of the reshaped AAM and its impacts on cell fate in PCa.

### 2.1. Glutamine

Glutamine, the most abundant amino acid in the bloodstream [13], is important for sustaining cancer cell growth and proliferation. Many proliferating cancer cells are addicted to glutamine as a crucial energy source and a critical biosynthesis precursor. An elevated glutamine metabolism has been associated with advanced PCa [14]. Glutamine and glutamic acid are markedly reduced in urine samples from PCa patients compared to those from healthy subjects [15,16].

The tricarboxylic acid (TCA) cycle serves as an essential pathway for cellular energy metabolism, occurring within the mitochondria, where the pyruvate generated from glycolysis is converted into acetyl-CoA and subsequently utilised to produce carbon dioxide, water and energy. In healthy prostate epithelial cells, the glucose-derived TCA cycle is disrupted at the citrate synthesis step due to the Zn^2+^ accumulated within the cells [17]. Hence, healthy prostate epithelial cells rely on glycolysis as their primary pathway for energy production; however, primary PCa exhibits a distinct glucose flux, characterised by enhanced oxidative phosphorylation and a limited rate of glycolysis [18]. This has been suggested to be the consequence of the decreased concentration of Zn^2+^ and the restored activity of aconitase [19,20]. Interestingly, as PCa develops, the contribution of glucose to the mitochondrial activity reduces, evidenced by increased aerobic glycolysis, known as the Warburg effect [21,22,23]. Despite the diminished glucose availability within the mitochondria, the activity of the TCA cycle remains elevated, as it is predominantly driven by glutamine metabolism. In the mitochondrion, glutamine is firstly converted into glutamate by glutaminase (GLS), which is then further processed by either glutamate dehydrogenase (GDH) or transaminase reactions to produce α-ketoglutarate (α-KG), an intermediate that feeds into the TCA cycle. The less catalytically active isoform of GLS1, KGA, predominates in early hormone-sensitive PCa. However, as the disease progresses into CRPC and small cell neuroendocrine carcinoma, the expressed GLS1 isoform shifts to a more enzymatically active and AR-independent GAC variant, enabling advanced PCa cells to preferentially utilise glutamine over glucose to fuel the TCA cycle [13,24]. This isoform switch forms the molecular basis for the high dependency of advanced PCa on glutamine rather than glucose. The robust enzymatic activity of the GLS1-GAC isoform enables tumour cells to channel greater amounts of glutamine into the TCA cycle, thereby enhancing the proliferative capacity of the cancer cells and driving more aggressive cellular behaviour [25]. By inhibiting GLS1 activity, particularly the GLS1-GAC isoform, it may be possible to effectively restrict the glutamine utilisation in PCa cells, thereby impeding their growth and proliferation.

### 2.2. Tryptophan

Tryptophan, an essential amino acid, serves as a precursor to serotonin and melatonin and contributes to nitric oxide synthesis, gut microbiota modulation and immune regulation, influencing immune responses and stress resilience. The significance of tryptophan in PCa has been extensively studied, with reports indicating that the tryptophan metabolism is significantly reduced in PCa patients compared to that in healthy controls [26,27].

Tryptophan metabolism plays a complex role in PCa metabolism, i.e., increased levels of tryptophan can either be beneficial or detrimental to PCa cells. High concentrations of tryptophan in prostate tumours lead to elevated levels of kynurenine [28], a terminal product of tryptophan metabolism which binds to and activates the aryl hydrocarbon receptor (AHR). The AHR is a transcription factor that regulates the expression of genes which promote cell growth, survival, DNA synthesis and cell cycle regulation, for example, *MYC* and *CCND1* (which encodes cyclin D1) [29]. When it is activated, the AHR translocates to the nucleus, where it binds to the DNA and initiates the transcription of downstream genes. Additionally, kynurenine suppresses T-cell activity, offering PCa cells an opportunity for immune evasion and thereby facilitating PCa cell survival and metastasis [30].

In contrast, it has also been demonstrated that tryptophan can be proapoptotic and antiproliferative for PCa cells. Several metabolites of tryptophan metabolism, such as 3-Hydroxyanthranilic acid, have been shown to inhibit PCa cell proliferation, potentially through mechanisms involving the suppression of protein and DNA synthesis. High tryptamine levels are known to induce apoptosis through evoking the intrinsic caspase cascade [31]. Further studies are still needed to dissect why dysregulation of the tryptophan metabolism pathway is both pro- and anti-PCa progression. Rebalancing the flux of tryptophan into different metabolites may be helpful for controlling cancer growth.

### 2.3. Leucine

Leucine is an essential amino acid that serves as a crucial energy source for tumour cell growth and survival. Many cancer cells rely on leucine to meet their rapid proliferation and metabolic demands. Inhibiting leucine uptake can often substantially reduce tumour cell proliferation and tumour growth [32]. In PCa research, leucine and its derivatives are often used as radiotracers for precise tumour localisation and for assessing the systemic tumour burden [33].

Leucine metabolism undergoes significant reprogramming in PCa. Leucine is catabolised within the cells to produce various metabolites, such as α-ketoisocaproate, that enter the TCA cycle. These metabolites provide energy and intermediate substrates for cancer cells [32,33]. Additionally, as a ketogenic amino acid, leucine is extensively consumed in metastatic PCa cells undergoing metabolic reprogramming, which induces a pronounced Warburg effect, further enhancing leucine’s glycolytic potential and supporting the maintenance of the stem-like phenotype of PCa stem cells [34].

Leucine metabolites are implicated in modulating the tumour microenvironment by influencing inflammatory responses and immune cell functions to impact on the progression of PCa [35]. Leucine is also a key activator of mTOR complex 1 (mTORC1), a main driver of mRNA translation in cancer cells. Please refer to Section 3.1 for more details.

### 2.4. Arginine

PCa is considered a typical immune-“cold” tumour, as PCa cells employ various mechanisms to evade immune surveillance, with intracellular arginine metabolism being one of the immunoregulatory factors. Arginine is a semi-essential amino acid that can be metabolised by arginase (ARG) to produce ornithine and urea or converted by inducible nitric oxide synthase (iNOS) to generate nitric oxide and citrulline [36,37]. ARGII and iNOS, respectively, are overexpressed in PCa tissue [38,39]. Enhanced arginine degradation inhibits the function of macrophages and T lymphocytes within the prostate tumour microenvironment and thereby impedes immune responses [37]. Evidence supports that inhibiting the activity of ARG and iNOS using specific inhibitors (N-monomethyl-L-arginine for iNOS and N-hydroxy-L-arginine for ARG) allows tumour-infiltrating lymphocytes to recover their responsiveness to tumour antigens [40].

Moreover, arginine starvation led to mitochondrial dysfunction in 22Rv1 and PC3 cells, evidenced by alterations in the mitochondrial morphology and the increased production of reactive oxygen species [41,42]. DNA damage and excessive autophagy are known to be associated with the silencing of nuclear-encoded mitochondrial genes, including those implicated in oxidative phosphorylation and nucleotide synthesis. Arginine acts as an epigenetic regulator of TEAD-4, a member of the mitochondrial transcription factor family that plays an important role in driving the expression of mitochondrial genes involved in oxidative phosphorylation, to enhance oxidative phosphorylation in PCa cells [43,44].

### 2.5. Methionine

Methionine is an essential amino acid involved not only in the initiation of protein synthesis but also in various cellular biochemical reactions, including biological methylation, antioxidation and polyamine synthesis. Methionine addiction, also known as the Hoffman effect, is one of the most characteristic metabolic reprogramming activities in cancer cells. This implies that cancer cells are unable to grow or proliferate under methionine deprivation, whereas non-cancerous cells can utilise homocysteine to replace methionine and remain unaffected [45]. Therefore, methionine restriction is considered to be a promising anti-cancer strategy [46]. Methionine restriction was able to markedly inhibit the growth of PC-3 and DU145 (androgen-insensitive) cells in vitro; meanwhile, LNCaP (androgen-sensitive) cells exhibited a weaker dependence on methionine [47], implying an even more increased demand for methionine in advanced, AR-null PCa. Furthermore, the depletion of methionine was shown to have apoptotic and anti-invasion effects on PC-3 and DU145 cells [9], and mitochondrial damage was the key factor in cell death, with the suppressed integrin/FAK pathway accountable for their inhibited invasion [9,48,49,50]. Clinical studies have demonstrated that for patients with advanced PCa and bone metastases, methionine deprivation therapy (MDT) resulted in a reduction in PSA levels [51,52]. These findings highlight the great therapeutic potential of MDT in the design of future anti-PCa therapies.

Metabolomics and transcriptome analyses have revealed significant alterations in the methionine metabolism in PCa tissue samples [53]. Methionine is able to be metabolised into S-adenosylmethionine (SAM) by methionine adenosyltransferases ((MATs) including MAT2α and MAT2β), which are encoded by *MAT2A* and *MAT2B*, respectively, in prostate tissues. *MAT2A* and *MAT2B* are highly expressed in PCa [54]. Overexpressed MAT can provide a survival advantage for cancer cells [55]. Two inhibitors of MAT2α, PF-9366 and AG-270, could significantly suppress VCaP cell growth in a spheroid assay, as well as enhancing the effectiveness of enzalutamide for CRPC in both in vivo and in vitro models [56]. Exogenous SAM could also be a promising PCa-suppressing agent, as evidenced by the pronounced reduction in PC-3 cell proliferation, migration and invasion after treatment with SAM [57]. Further investigations have elucidated the potential mechanisms of its anti-cancer effect on PCa cells. For example, SAM inhibited the activation of ERK and STAT3 in both PC-3 and DU145 cells, and the miR-34a/b-MAT2α/MAT2β signalling axis could play a role in this regulation [54,58]. SAM could also downregulate the transcription of oncogenes by acting as a methyl group donor for DNA methylation, thereby preventing PC-3 cell growth [59,60].

### 2.6. Histidine

Histidine is one of the least abundant amino acids in the human body. Apart from its direct participation in the process of protein synthesis, it can also be converted into urocanate for glutamate metabolism, while the small remaining portion is further metabolised into carnosine, histamine and other histidine derivatives. Notably, histamine plays a key role in inflammation and immunity [61]. In mouse prostate tissues, a high-fat diet induced the expression of *Hdc*, which encodes histidine decarboxylase, and led to a significant increase in histamine levels and cytokine expression [62]. As a receptor antagonist for histamine signalling, fexofenadine alleviated the inflammation stimulated using histamine in a murine PCa model and inhibited tumour growth in vivo, implying that targeting histamine could be a novel strategy against obesity-induced prostate carcinogenesis.

### 2.7. Serine

Serine, a non-essential amino acid, can be acquired from extracellular sources via the serine–lysine–cysteine–threonine transporter (ASCT) or synthesised endogenously from glycolytic intermediates, such as 3-phosphoglycerate or glycine. Serine is fundamental to the growth and proliferation of cancer cells [63]. Although the human body can synthesise most serine through biosynthetic pathways [64], rapidly proliferating cancer cells still require external serine sources to meet their high metabolic demands. The plasma serine levels are significantly elevated in PCa patients, particularly those with a Gleason grade higher than 6 [65,66], and treatment with ASCT inhibitors has been shown to suppress the growth of LNCaP and 22Rv1 cells [67]. However, due to real-life challenges such as unavoidable dietary sources of serine and the fact that the transport of other amino acids (i.e., lysine, cysteine and threonine) also relies on ASCT, exogenous serine restriction remains unrealistic as an anti-PCa strategy [68].

Serine metabolism is intricately connected to various critical metabolic activities, attracting considerable interest from cancer researchers. For example, the Serine–Glycine–One-Carbon (SGOC) metabolic network is essential for the biosynthesis of proteins, lipids and nucleic acids, which are required for cell growth and proliferation. Upregulation of the SGOC network is crucial for sustaining rapid cancer cell proliferation in several malignancies, including neuroendocrine PCa (NEPC) [69]. Recent studies have revealed several key drivers of PCa within the SGOC network, including phosphoglycerate dehydrogenase (PHGDH) and serine hydroxymethyltransferase (SHMT1/2) [70,71]. PHGDH, the initial and rate-limiting enzyme in the SGOC pathway, is highly expressed in breast cancer [72], bladder cancer [73] and PCa [69]. It is also implicated in the drug resistance mechanisms across various cancers [74]. Wang et al. demonstrated that PHGDH knockdown could suppress the growth of enzalutamide (AR antagonist)-resistant CRPC cells and restore their sensitivity to enzalutamide [75]. SHMT1/2, a set of isoenzymes responsible for catalysing the conversion of serine into glycine in the cytoplasm and mitochondria, promoted tumorigenesis in breast and ovarian cancer and PCa [76]. The expression of the *SHMT2* gene was upregulated in primary prostate tumours [77]. SHMT2 promoted the proliferation and metastasis of PCa by affecting energy metabolism and promoting the epithelial–mesenchymal transition (EMT). Diminished SHMT2 expression in invasive PCa cells resulted in enhanced cell viability, migration and upregulation of genes related to the EMT through MAPK pathway activation [78]. Additionally, IL-6-stimulated inflammatory responses evoked oxidative stress and increased lactate fermentation via the STAT3/SHMT2/PKM2 pathway during the early stage (Gleason score: 6) of PCa. As the disease advances, persistent inflammatory states lead to oxidative stress, activating the STAT3/HIF-1α/PKM2 axis to sustain the Warburg effect, a phenomenon corroborated by comparing PCa tissue samples from patients with different Gleason scores [79].

### 2.8. Glycine

Glycine plays a crucial role in mRNA translation and the synthesis of small biomolecules such as creatine, purines and glutathione. The first metabolite profiling of cancer derived from the NCI-60 cancer cell line screen revealed that cancer cells exhibit significant dependency on glycine [80]. Remarkable differences in the serum and urine glycine levels were observed in PCa patients compared with healthy individuals [81,82,83,84,85]. Rapidly proliferating cancer cells exert a high demand on glycine metabolism [80,86], although this may be an indirect effect owing to the conversion of glycine into serine [63]. Recent studies have indicated that the mitochondrial glycine cleavage system (GCS) plays an important role in cancer [87,88,89,90], providing new insights into the effects of dynamic changes in glycine metabolism in cancer progression. GCS is the primary enzymatic system for glycine catabolism in the human body [91], wherein *GLDC*, which encodes the glycine decarboxylase protein, has been found to be significantly upregulated in various cancers, including PCa [92,93,94]. The overexpression of *GLDC* in 22RV1 cells enhanced glycolytic metabolism and played a critical role in cell invasion, metastasis and immune evasion [95].

Aminoacyl-tRNA synthetases (ARSs) are essential enzymes in the translation process that convert genetic information into proteins. They are responsible for attaching amino acids to their corresponding tRNA molecules to form aminoacyl-tRNAs. An increasing body of evidence indicates that ARSs are not only pivotal for protein biosynthesis but also play additional roles in other physiological and pathological cellular processes [96]. For instance, the aberrant expression or post-translational modification of ARSs can affect cancer angiogenesis, invasion and metastasis [97,98]. Elevated levels of GARS, HARS, WARS and KARS have been detected in PCa cells in response to androgens [99,100]. GARS expression was positively correlated with PCa cell cycle progression, proliferation, migration and their ability in immune escape [101,102].

**Figure 1:**
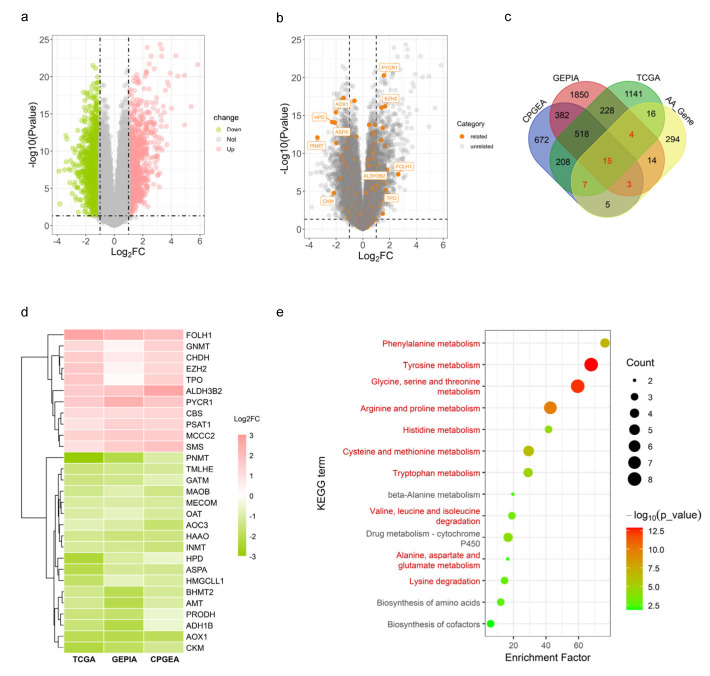
Differentially expressed genes related to AAM in PCa. (**a**) The volcano plot illustrates differentially expressed genes that were identified from the TCGA RNA-seq data of PCa patients. (log_2_FoldChange (log_2_FC) ≥ 1, *q* < 0.05). The green dots indicate genes that are downregulated in PCa tissues, while the red ones indicate genes that are upregulated in the disease. (**b**) The same as A but genes associated with AAM are coloured in orange. The top 5 most up- or downregulated AAM-related genes are labelled. (**c**) Differently expressed genes implicated in AAM (AA_DEGs) in primary PCa tissues were identified according to the GEPIA [103], CPGEA [104] and TCGA [105] databases, and a total of 29 genes were identified which were differentially expressed in at least two databases. (**d**) Heatmap illustration of the 29 AA_DEG genes mentioned in (**c**). (**e**) KEGG enrichment analysis of the AA_DEGs.

While focusing on these metabolic changes in PCa, we noticed that the dysregulation of certain genes’ expression is involved in alterations in AAM paradigms (Figure 1 and Table 1). In the following sections, we will extend the discussion of the aberrant transcriptional regulation of key genes implicated in AAM, which can be leveraged to design potential anti-PCa treatment strategies.

**Table 1:** Functional annotation of the differentially expressed AAM-related genes in PCa.

Amino Acid	Gene Symbol	Full Name	Biological Function	Alteration in PCa	Effects on PCa	Reference
Arginine	*CKM*	Creatine kinase, M-type	Catalyses the reversible transfer of a phosphate group between ATP and various phosphogens	↓ ^a^	Decrease in both trancription and protein levels; high expression was correlated with prolonged relapse-free survival	[106]
Aspartate	*FOLH1*	Folate hydrolase 1	Acts as a glutamate carboxypeptidase and plays a key role in folate metabolism by breaking down folate and folate derivatives	↑ ^b^	Known as the prostate-specific membrane antigen and highly expressed; a diagnostic biomarker	[107,108]
Leucine	*MCCC2*	Methylcrotonyl-CoA carboxylase subunit 2	Engaged in leucine and isovaleric acid catabolism	↑	As an androgen-regulated gene; involved in carcinogenesis by upregulating GLUD1 in the GLUD1-p38 MAPK signalling axis	[109,110]
Lysine	*EZH2*	Enhancer of zeste 2 polycomb repressive complex 2 subunit	Responsible for trimethylating histone H3 on lysine 27, i.e., yielding H3K27me3, a histone modification that represses gene transcription	↑	Overexpression promoted tumour proliferation, metastasis, drug resistance and poor prognosis	[111,112,113,114]
	*MECOM*	MDS1 and EVI1 complex locus	Encodes an oncoprotein (transcription factor) known as Evi-1, regulating the gene expression during hematopoiesis and development	↓	Overexpression contributed to docetaxel resistance in PC3 cells	[115]
Proline	*OAT*	Ornithine aminotransferase	Catalyses the reversible interconversion of L-ornithine and 2-oxoglutarate into L-glutamate semialdehyde and L-glutamate	↓	Repressed by AR knockdown in C4-2B cells	[116]
	*PRODH*	Proline dehydrogenase 1	A mitochondrial protein that catalyses the oxidation of proline and its conversion into P5C in a FAD-dependent manner	↓	Upregulation in both mRNA and protein levels; overexpression promoted PCa tumour growth and reduced T-cell infiltration in mice	[117]
	*PYCR1*	Pyrroline-5-carboxylate reductase 1	One of the isoforms of the pyrroline-5-carboxylate reductase, which is involved in proline biosynthesis	↑	*PYCR1* knockdown suppressed the growth of DU145 and PC-3 cells	[118,119]
Tryptophan	*INMT*	Indolethylamine N-methyltransferase	Involved in the methylation of indolethylamines, such as tryptamine and serotonin, converting them into N-methylated derivatives	↓	Low expression in primary PCa but elevated in CRPC.; *INMT* knockdown in DU145 cells was inhibitory to cell proliferation	[120,121,122]
Multiple	*AOX1*	Aldehyde oxidase 1	Exhibits a broad substrate specificity and involved in cell redox homeostasis by regulating the production of reactive oxygen species	↓	Hypermethylated and downregulated; overexpression inhibited the proliferation and invasion of PC-3 cells	[123,124]
	*ASPA*	Aspartoacylase	Crucial to the breakdown of N-acetylaspartate into aspartate and acetate	↓	Inhibited the phosphorylation of LYN and suppressed the proliferation, migration and invasion of PC-3 cells	[125]
	*CBS*	Cystathionine beta-synthase	The rate-limiting enzyme in the first step of the transsulfuration pathway, catalysing the conversion of homocysteine into cystathionine; SAM is required for the allosteric activation of its homotetramer	↑	Elevated in the early stages but drops during tumour metastasis	[126]
	*GNMT*	Glycine N-methyltransferase	Catalyses the transfer of a methyl group from SAM to glycine, producing S-adenosylhomocysteine and sarcosine	↑	Regulated in an AR-dependent manner and contributed to the survival of PCa cells	[127,128,129]
	*MAOB*	Monoamine oxidase B	Located at the mitochondrial outer membrane and involved in the breakdown of monoamines, including neurotransmitters like dopamine and serotonin	↓	Decreased expression could enhance the proliferation of PC-3 cells and was associated with a poor prognosis in PCa patients	[130,131]
	*SMS*	Spermine synthase	Catalyses the production of spermine from spermidine and decarboxylated SAM	↑	Sensitive to androgen exposure	[132,133,134]

a: ↑ indicates that the gene is upregulated in PCa; b: ↓ indicates that the gene is downregulated in PCa.

## 3. Signalling Pathways Affected by an Altered AAM in PCa

### 3.1. mTOR

Amino acids are the cornerstone of protein synthesis. Distinct amino acids utilise a series of signal transduction mechanisms to modulate the mRNA translation machinery. In mammalian cells, mTOR serves as a central hub for sensing environmental stimuli, including amino acids, to promote protein synthesis and modulate anabolic pathways, thus governing cell growth and proliferation [135]. mTOR is a serine/threonine protein kinase that acts as the catalytic subunit of two distinct protein complexes, mTORC1 and 2. Amino acids can activate mTORC1 via RAG GTPase-dependent and -independent pathways. RAGs facilitate the recruitment of mTORC1 to the lysosomal surface, where it can be activated by its upstream activator Rheb-GTP [136]. Certain amino acids, such as leucine and arginine, act as positive regulators of mTORC1 by evoking the activation of RAGs [137,138]. Cells can also directly sense changes in amino acid levels through specific intracellular sensors, including the leucine sensors Sestrin2 [139], SAR1B [140] and LARS [141] and the arginine sensors CASTOR1 [137] and SLC38A9 [142]. It is noteworthy that many amino acid transporters are multi-functional; they can serve as receptors for sensing amino acid signals, as well as becoming the gateways for amino acid entry through the plasma membrane. For example, leucine is transported into cells via the L-type amino acid transporter (LAT) family. The inhibition of LAT proteins using the leucine analogue BCH reduced mTORC1 activation and suppressed the growth of PCa cells (LNCaP, PC-3 and DU145) [143,144]. Interestingly, the primary LAT protein used by PCa cells to intake leucine can alter according to the disease progression. In localised prostate tumours, PCa cells predominantly rely on LAT3 (SLC43A1) for leucine transport. However, as the cancer progresses to CRPC, the LAT3 expression decreases, whereas LAT1 (SLC7A5) expression increases and becomes the main channel for leucine uptake [144,145]. Supporting this notion, Wang et al. reported that knocking down LAT1, but not LAT3, in androgen-insensitive PC3 cells significantly inhibited mTORC1 activation [143]. Consistent with this notion, the LAT1-specific inhibitor JPH203 also showed potential as an effective treatment for CRPC [146,147].

Glutamine is an amino acid that governs mTORC1 activity via a RAG-independent route, featuring a distinctive and intricate regulatory mechanism. Increased glutamine dependence is commonly observed during the progression of PCa [7], and elevated glutamine levels have been shown to increase the mTORC1 activity in C4-2 cells [148]. The primary transporter of glutamine in PCa cells is ASCT2 (SLC1A5). Inhibition of ASCT2 using its competitive inhibitor BenSer and GPNA has been shown to effectively block the phosphorylation of mTORC1 downstream targets in PC-3 cells; however, this effect was not observed in LNCaP cells [149]. ATP is a crucial energy source for mTOR activation [150], and AMPK (adenosine monophosphate-activated protein kinase) is the principal energy sensor in cells and a negative regulator of mTORC1 [151]. Glutamine can serve as an energy source for increasing intracellular ATP levels and thus indirectly activate mTORC1 by inhibiting AMPK in HMVP2 (murine PCa) cells [152]. In summary, distinct amino acid signalling cues can stimulate mTORC1 through different mechanisms to control protein synthesis and cell fate. Although mTOR inhibitors have been ineffective against PCa in clinical trials so far due to high toxicity and the activation of feedback mechanisms [153], many other amino acid–mTORC1 signalling pathway components may still be suitable therapeutic targets for anti-PCa treatment. For example, Hsueh et al. found that a lack of arginine also inhibited mTOR signalling in PCa cells [154]. Zhao et al. revealed the important role of VWCE in the mTOR response to amino acid starvation in PC-3 cells [155]. For a greater understanding of the signalling process, there is certainly a fertile research area for future studies to explore.

### 3.2. GCN2

**Figure 2:**
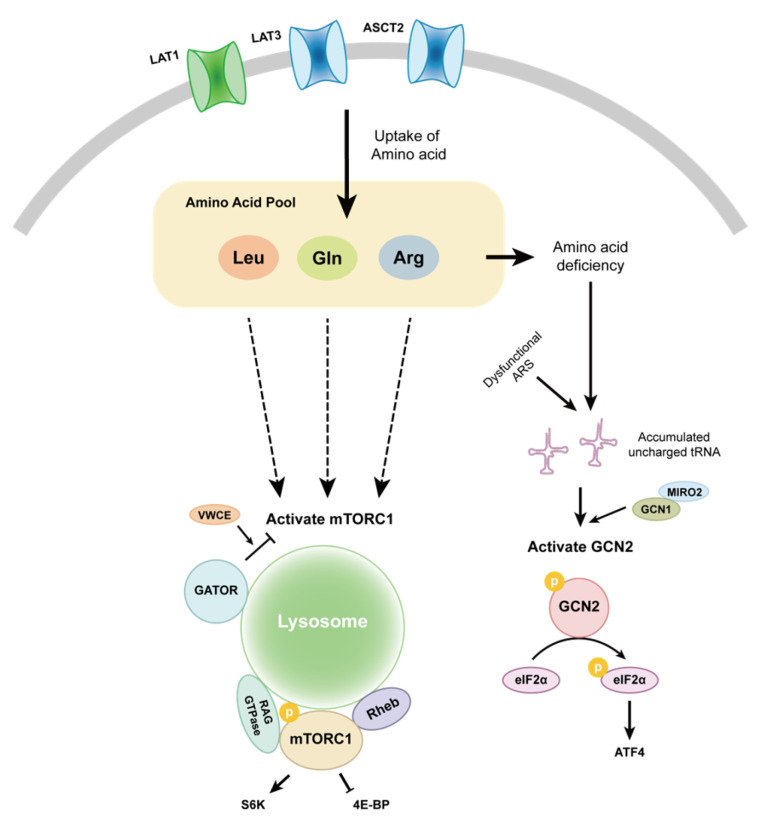
The mechanism of amino acid signal sensing in PCa. PCa cells transport amino acids through various membrane transporters such as LAT1, LAT3 and ASCT2. Leucine, glutamine and arginine can regulate the mTORC1 signalling pathway via distinct mechanisms. Amino acid deprivation or the reduced activity of ARSs can stimulate GCN2, leading to the phosphorylation of eIF2α (eukaryotic translation initiation factor 2α) and the subsequent upregulation of *ATF4* (Activating Transcription Factor 4) expression.

GCN2 is a serine/threonine protein kinase in eukaryotic cells that plays a pivotal role in amino acid sensing and stress responses. Unlike mTORC1, which relies on specific amino acid sensors, GCN2 responds to amino acid deficiency by binding to uncharged tRNAs, and therefore its activation is not constrained by a specific type of amino acid [156], although recent studies have also suggested that GCN2 can be activated in a manner independent of deaminated tRNAs [157]. The activating mechanisms of mTORC1 and GCN2 are illustrated in Figure 2. Once activated, GCN2 phosphorylates eIF2α, leading to global inhibition of protein synthesis while also promoting the translation of specific genes, such as the transcription factor *ATF4*, which further assists the cells in adapting to amino acid scarcity. Increased expression and phosphorylation levels of GCN2 have been observed in various cancer tissues, including those from colon, lung and breast cancer [158] and PCa [159]. The GCN2-eIF2α-ATF4 signalling axis was found to support cancer cell growth and proliferation by promoting the expression of amino acid transporters and activating autophagy [160]. Amino acid deprivation and uncharged tRNAs were able to activate GCN2 in LNCaP cells, and GCN2 deletion or inhibition reduced PCa cell proliferation in vitro and in vivo [161]. GCN1 (general amino acid control non-depressible 1), as an upstream activator of GCN2, also plays an important role in initiating downstream signalling cascades in response to cellular stress. Furnish et al. noted that MIRO2 engaged in GCN2 sensing of amino acid deficiency by interacting with GCN1 in PCa cells [159], demonstrating MIRO2 as a potential target for GCN2 inhibition. It is noteworthy that after the treatment of PCa cell lines, such as LNCaP, PC3 and 22Rv1, with GCN2iB (a specific competitive inhibitor of GCN2), the growth of these cells was impeded, and a series of genes engaged in AAM were downregulated at both the transcription and translation levels, such as *ASNS* (induced by ATF4) and a range of SLC gene family members, including *SLC7A11* (encoding xCT), *SLC7A5* (encoding LAT1) and *SLC3A2* (encoding 4F2) [161]. Thus, PCa cells utilise AR- and ATF4-mediated transcriptional pathways to modulate the expression levels of amino acid transporters to sustain a sufficient intracellular amino acid supply. In summary, mTORC1 and GCN2 are two signalling sensors of amino acid availability for controlling protein synthesis, providing the building blocks for PCa cell growth and proliferation. Given the issues with mTOR-inhibitor-related PCa clinical trials, it would be intriguing to consider GCN2 as an alternative anti-PCa treatment target in future clinical studies.

## 4. Concluding Remarks

AAM is a core component of cellular metabolism, participating in various physiological processes, from protein synthesis to energy production, and is closely linked to maintaining cellular homeostasis and normal cell function. The universality and significance of metabolic reprogramming in cancer cells have increasingly been recognised; thus, focusing on abnormal changes in AAM holds promise for providing new perspectives and potential targets for cancer diagnosis and treatment. In this review, we discussed how the AAM landscape is altered in PCa. Metabolic patterns of certain amino acids such as glutamine, leucine, arginine, methionine and serine are remodelled during the progression of PCa, providing essential nutrients and energy for PCa cell growth and survival. We further explored how amino acids leverage the mTORC1 and GCN2 signalling pathways to stimulate mRNA translation, also in the context of PCa.

Based on these findings, AAM pathways hold promise as therapeutic targets for PCa, and related research has provided a crucial theoretical foundation for developing new treatment strategies for PCa. However, the precise role of many genes related to AAM that are abnormally expressed in PCa (AA_DEGs; see Figure 1) still awaits further investigation. Understanding the regulatory mechanisms of these genes could help elucidate the pathological processes of PCa, particularly in its progression to more refractory phenotypes. These genes may serve as potential therapeutic targets for PCa. Table 1 summarises all of the AAM-related genes covered in this review and their impacts on PCa. On a cautionary note, cell and thus AR specificity can easily be ignored when interpreting amino-acid-signalling- and PCa-related data since different PCa cell lines exhibit distinct levels and isoforms of AR and would therefore have different impact on related signalling pathways. Similarly, changes in the PCa microenvironment under different PCa subtypes (e.g., AR-dependent/independent, CRPC, NEPC) may significantly influence the outcomes of treatment strategies targeting AAM pathways. Therefore, we suggest that when it comes to the choice of the experimental models, future studies should consider making conclusions not only based on established PCa cell lines and murine models but rather also relying on more sophisticated PCa patient sample models (e.g., patient-derived tumour explants and patient-derived xenografts) to consolidate their findings. We anticipate that further elucidation of the molecular mechanisms behind the abnormal and tumour-type-specific AAM in PCa patients will achieve more breakthroughs and advancements for PCa treatment.

## Data Availability

The datasets analysed during the current study are available in The Cancer Genome Atlas (TCGA) repository at https://portal.gdc.cancer.gov/ (accessed on 19 August 2024), Gene Expression Profiling Interactive Analysis (GEPIA) repository at http://gepia.cancer-pku.cn/ (accessed on 22 July 2024) and Chinese Prostate Cancer Genome and Epigenome Atlas (CPGEA) at http://www.cpgea.com (accessed on 27 October 2021, original publication here: https://doi.org/10.1038/s41586-020-2135-x).
